# Clinical insights into the management of sleep disturbances within cancer care: a qualitative analysis

**DOI:** 10.1007/s00520-026-10478-4

**Published:** 2026-02-27

**Authors:** Sam Adams, Timothy D. Clay, Mitchell Turner, Christopher Kueh, Kelly Moes, Travis Cruickshank

**Affiliations:** 1 School of Medical and Health Sciences, Edith Cowan University, Joondalup, WA 6027 Australia; 2 Centre for Precision Health, Edith Cowan University, 270 Joondalup Drive, Joondalup, WA 6027 Australia; 3 Department of Oncology, St John of God Subiaco Hospital, Subiaco, WA 6008 Australia; 4 School of Arts and Humanities, Edith Cowan University, Mount Lawley, WA 6050 Australia; 5 Centre for Culture and Technology, Curtin University, Bentley, WA 6102 Australia; 6 Perron Institute for Neurological and Translational Sciences, Perth, WA 6009 Australia

**Keywords:** Sleep disturbance, Clinician management, Cancer care

## Abstract

**Purpose:**

The present study aimed to gain a deeper understanding of how oncology healthcare professionals’ (HCPs) manage sleep disturbances.

**Methods:**

Semi-structured interviews were conducted with 10 oncology HCPs (medical oncologists, oncology nurse practitioners, clinical nurse consultants) working in metropolitan Perth, Western Australia. Reflexive thematic analysis was used to understand oncology HCPs’ perspectives on treating sleep disturbances, explore challenges and barriers to their management, as well as opportunities for improvement.

**Results:**

Four main themes were developed: (1) sleep disturbances are underreported and underassessed, (2) poor sleep can be difficult to manage, (3) limited capacity to address sleep issues, and (4) opportunities for the future.

**Conclusion:**

Understanding the current clinical management practices used by oncology HCPs to manage sleep disturbances, along with their perceived opportunities to improve the management of sleep in cancer care is crucial. Efforts to implement these opportunities should focus on co-design with relevant stakeholders at all levels.

**Trial registration:**

Registry: ANZCTR.org.au, TRN: ACTRN12622001035718, registration date: July 25, 2022.

**Supplementary Information:**

The online version contains supplementary material available at 10.1007/s00520-026-10478-4.

## Introduction

Sleep disturbances are common amongst individuals with cancer receiving chemotherapy and are associated with greater symptom burden [1], disease progression [2], and reduced health-related quality of life [3–5]. Ineffectively managed, sleep problems persist into survivorship and have long-term physical and psychological consequences [6, 7]. Despite the prevalence and impact on health, sleep problems are often underreported, under-recognised and undertreated within cancer care [8–10].

The aetiology of sleep disturbances is complex and multifaceted. Poor sleep may be attributable to cancer-specific factors (tumour pain, dyspnoea), medications and treatment-related side effects (corticosteroids, hormonal fluctuations, fatigue), as well as psychological factors (anxiety and depression) [11, 12]. Maladaptive behaviours including increased daytime napping [5] and reduced exposure to daytime light [13] may also drive sleep disturbances. Effectively managing and treating these issues presents a challenge for oncology healthcare professionals (HCPs).

Despite clinical practice guidelines existing for the management of sleep disturbances and/or sleep disorders specific to cancer [12, 14, 15], sleep may not be screened in routine care. In a survey of cancer survivorship programs in the United States, 56% reported screening fewer than one in four patients for insomnia and almost two-thirds of programs indicated that less than 25% of their patients received optimal care for sleep issues [16]. Lack of training or education around sleep, clinical experience, time constraints, limited access to sleep-specific resources and HCPs present as barriers to effectively managing sleep disturbances within cancer care [17].

Although the prevalence of sleep disturbances in individuals with cancer is well documented, as are different treatments and their effectiveness, little is known about how oncology HCPs manage sleep disturbances. Jeon et al. [17] surveyed 73 HCPs treating brain tumour patients and found most felt limited in managing sleep disturbances due to lacking specific sleep knowledge, training, and time. The extent to which these issues permeate the broader cancer care field is not well known.

The present study aimed to gain a deeper understanding of how sleep disturbances are managed by oncology HCPs working in metropolitan Perth, Western Australia, with a specific focus on sleep disturbances in individuals treated with chemotherapy, where sleep disturbances commonly manifest and left unmanaged negatively impact health and social outcomes.

## Methods

### Study design

A qualitative descriptive design was undertaken to explore how oncology HCPs manage sleep disturbances. This approach facilitates the generation of rich data from participants’ first-hand experiences, which can be used to comprehensively understand a phenomenon whilst being theoretically and interpretively flexible [18, 19]. This study adhered to the Standards for Reporting Qualitative Research (SRQR) guidelines [20] (see Supplementary File 1).

### Participants

Ethical approval was granted by the Human Research Ethics Committees at St John of God Health Care (#1907) and Edith Cowan University (2022–03777-CRUICKSHANK). Interested medical oncologists, clinical nurse consultants, and oncology nurse practitioners that practice in metropolitan Perth, Western Australia were approached via email to participate in the present study.

### Procedure

Participants were sent an email which detailed an overview of the study and an invitation to participate. Once consent was obtained, the study author (SA) organised a subsequent interview. Interviews took place between April and May 2024 using Microsoft Teams.

Three of the study authors (SA, KM, TC) developed an interview guide (see Supplementary File 2). The semi-structured interview questions were intended to facilitate an inductive exploration into the oncology HCPs’ experiences in managing sleep issues. Qualitative analyses were undertaken with Microsoft Excel and NVivo 12 software. All derived themes were reviewed by multiple authors to best ensure accuracy.

### Reflexive thematic data analysis

Reflexive thematic analysis (RTA) was used to understand oncology HCPs’ perspectives on treating sleep disturbances, explore challenges and barriers in care, and opportunities for improvement. Recommendations as outlined by Braun and Clarke [21] were followed for the analysis. Transcripts were first read and re-read by two of the study authors (SA and KM) to facilitate familiarisation. Codes were then systematically generated independently by the two authors, predominantly using an inductive approach with minimal deductive analyses. The initial themes were then generated, developed, and reviewed by the study authors (SA, KM, TC). Following this, the first author refined, defined, and named the themes; this process was iterative throughout writing the report. The results are reported below.

## Results

### Participants

Twenty-four invitations were sent to oncology HCPs that practice in metropolitan Perth, Western Australia. Ten individuals expressed interest and consented to participate. Participant characteristics are provided in Table 1.

**Table 1:** Characteristics of 10 oncology HCPs

Characteristic	*n* (%)
Age (years; mean (SD))	44.70 (± 5.97)
Sex	
Male	3 (30%)
Female	7 (70%)
Occupation	
Consultant medical oncologist	6 (60%)
Oncology nurse practitioner	2 (20%)
Clinical nurse consultant (cancer services)	1 (10%)
Clinical nurse consultant (palliative care)	1 (10%)
Years experience in cancer care (years; mean (SD))	15.66 (± 8.60)
Practice location	
Private hospital	3 (30%)
Public hospital	6 (60%)
Specialist centre	1 (10%)
Interview length (minutes; mean (SD))	15.58 (± 3.30)

### Thematic analysis

Four main themes and seven subthemes were developed and are reported below.

### Theme 1: sleep disturbances are underreported and under-assessed

The first theme describes how sleep disturbances are often underreported and underassessed in this population.

## Sleep issues are not prioritised

Oncology HCPs noted that patients do not often report sleep issues to their treating clinicians. “Patients don’t raise it. You have to ask them specifically” (Oncology Nurse Practitioner [1]). Clinicians believed it was their responsibility to initiate conversations regarding sleep. “If you leave it to them… they might not bring it up because they think… it might be a small issue. So I think it’s incumbent on the clinician to target out and ask” (Medical Oncologist [4]).

Clinicians have several competing priorities and unless they suspect a patient may be at a higher risk of sleep disturbances or include general questions about sleep in their clinical examinations, it is not routinely screened. “It’s something that I don’t specifically ask for unless a patient divulges… issues with sleep” (Medical Oncologist [3]).

## Sleep assessments are rarely used

Oncology HCPs described rarely using formal sleep assessment tools, instead relying on informal, subjective questioning during clinical consultations. These questions were often general and typically asked if a patient appeared at higher risk of sleep problems. “I don’t use any sort of tools… we just ask general questions… how are you sleeping?” (Medical Oncologist [1]). Another clinician reported tailoring questions to each patient rather than using structured tools. “It’s about… looking at the patient in front of you and asking the questions” (Medical Oncologist [4]). One clinician described the impact of sleep assessments on the flow of conversation. “It impacts the conversation. It’s not an easy free flowing conversation… patients sometimes find… how do you measure sleep, zero to ten… we’re used to doing it with pain… but with sleep… it’s harder to actually quantify” (Clinical Nurse Consultant, Palliative Care [1]).

Sometimes, sleep assessments are used if required for a sleep study or referral to a sleep or respiratory physician.

I’ve got some suspicion that they might have sleep disordered breathing separate to their cancer treatment… I’ll do a STOP-Bang questionnaire or an Epworth sleepiness scale… on the basis… referral to a sleep or respiratory physician, to go looking for sleep apnoea… those scales are required sometimes to do an upfront sleep study. So that’s on the basis of a particular referral requirement Medical Oncologist [5].

### Theme 2: poor sleep can be difficult to manage

## Sleep disturbances are complex

The second theme explores the complexity of sleep disturbances and their challenges in cancer care. The following excerpt was communicated by an oncology nurse practitioner:

Some patients, it might be their sleep hygiene… napping during the day… drinking a lot of water… up and down all the time with going to the toilet. Some of them it’s a psychological thing… at night they can’t get their mind off their diagnosis… prognosis. For some people, it’s purely medication-based. Oncology Nurse Practitioner [1].

This quote illustrates the multifactorial nature of sleep disturbances in this population, which may be a combination of treatment-related, psychological, and behavioural factors.

Treatment-related factors, such as corticosteroid usage and hormonal fluctuations, were noted to impact sleep. Corticosteroids were frequently described as adversely affecting sleep in this population. “I mean, the biggest, the biggest driver of sleep disturbance in our population is the need to use corticosteroids” (Medical Oncologist [5]). Hormonal fluctuations induced by chemotherapy were also discussed. “Vasomotor symptoms related to endocrine dysfunction … causing them hot flushes that stop them from sleeping” (Medical Oncologist [5]).

Furthermore, co-occurring symptoms such as pain, fatigue, and anxiety were identified as contributing to sleep disturbances. “Looking at other reasons why they might not be sleeping, … depression or anxiety… rumination about their diagnosis, or anxiety about death, or treatment side effects” (Medical Oncologist [5]).

## Need to determine the underlying issue

Oncology HCPs recognised the importance of determining the underlying cause of poor sleep where possible, although it could be difficult to determine. “It might be related to steroids, … cancer anxiety… pain… and so making an assessment of what the primary cause is and then trying to treat that underlying cause” (Medical Oncologist [6]). One clinician noted that sleep disturbances are often secondary to another symptom. “Sleep disturbance is a symptom of something else being wrong… it’s often reactive to something else… drug side effect… mental health issue… secondary to a physical symptom” (Medical Oncologist [5]).

## Treatment effectiveness varies

Oncology HCPs perceive treatments to have varying effectiveness amongst their patients. Sleep hygiene was regularly discussed with patients, although it was noted to have mixed success. “I do think sleep hygiene is really critical, but I feel like it’s not the be-all and end-all” (Oncology Nurse Practitioner [1]). Referral to psychological services was considered for patients with poor sleep and significant psychological and emotional distress. “We also have psychologists… if it was an anxiety… mood issue… we’d refer them to a psychologist… we have mixed responses, some people find it… quite useful, some… don’t think… would be much value for them” (Medical Oncologist [2]). Where symptoms such as pain were impacting sleep, clinicians found palliative care services to be effective in treating sleep. “For people with complex pain symptoms, I refer them to palliative care, and they’re very good at addressing most of these issues, including sleep” (Medical Oncologist [1]).

Pharmaceutical sleep aids were the most common treatment utilised by oncology HCPs to manage sleep disturbances. Olanzapine and mirtazapine were preferred over benzodiazepines due to their more favourable side effect profile. “Olanzapine is probably the best option that I’ve found. Occasionally, you get the problem that it is too sedating” (Medical Oncologist [5]). Most clinicians prescribed melatonin, although its effectiveness varied. “Melatonin’s a little bit hit and miss” (Medical Oncologist [5]).

Some clinicians reported using benzodiazepines, although this was typically prescribed to select patients and intermittently used. “Generally we are taught not to be prescribing too many benzodiazepines. I personally don’t like prescribing benzodiazepines… I limit how much I prescribe… Also… patients… don’t want to be addicted… we don’t want any dependence developing… so have to sort of find a fine line between the two” (Medical Oncologist [1]). Furthermore, clinicians noted concerns with prescribing too many different medications. “Polypharmacy is a challenge … patients are already on multiple medications, so if you’re trying to look for pharmacological solutions there’s interactions… our population’s ageing… standard sleep medications come with risks… I think that choosing the safe method to help them with their sleep… is challenging in the patient population that we’re increasingly seeing” (Medical Oncologist [3]).

### Theme 3: limited capacity to address sleep issues

## Education gaps, time constraints, and limited access to resources

The third theme highlights the challenges in managing poor sleep at an individual and system level. Collectively, oncology HCPs commented on a lack of knowledge and education about sleep as a challenge. “Not something… we really are taught about… it’s something… pick up along the way… pick up your habits in managing sleep disturbance from clinical practice” (Medical Oncologist [5]).

Time constraints were the most frequently described challenge faced by clinicians. “We’re so busy as oncologists, that it’s often not at the forefront of our mind and addressing it takes time” (Medical Oncologist [5]). Clinicians also noted that due to time constraints, sleep was not often prioritised and as such may not be addressed. “It’s not regarded as a first order issue… gets pushed down the list… it’s not… prioritised” (Medical Oncologist [2]). Where clinicians may have the time to implement a treatment, lack of follow-up was another key challenge. “I’m not always the one that follows them up, so it’s a bit frustrating really. So I might implement something, but then not see them for another couple of visits. So it’s hard to know” (Oncology Nurse Practitioner [1]).

Limited access to resources was also noted as a barrier to managing sleep disturbances. Where referral to psychological services was warranted, these services were described as costly and limited. “Psychologists are very good… very resource limited. Unfortunately, there’s not enough money for psychologists to go around” (Medical Oncologist [6]). Another key challenge raised was the model of care. “We’re treatment-focused, and in a surveillance population… we’re not in a survivorship clinic model, nor do we have capacity to do that” (Oncology Nurse Practitioner [2]).

Clinicians also noted limited accessibility to effective treatment options. “We lack good options that are not habit forming, or don’t have significant side effects” (Medical Oncologist [5]).

### Theme 4: opportunities to improve the management of sleep disturbances within cancer care

## Encourage patients to self-report, integrate general practitioner (GP) and improve access to resources

The fourth theme explores perspectives for improving the management of sleep disturbances in cancer care. Clinicians actively encouraging patients to report sleep issues was perceived as a key opportunity. “I just encourage patients to keep reporting” (Oncology Nurse Practitioner [1]). Conversely, clinicians recognised the importance of screening and assessing for sleep issues with patient-reported outcome measures (PROMs). “Resourcing towards understanding… the extent of the problem in terms of PROMs… would be helpful” (Medical Oncologist [3]). Although clinicians agreed that increased screening of sleep disturbances would be useful, it was noted that not having the capacity or resources to act on the findings may be more harmful. “When you identify something and you don’t have capacity to deal with it, have you opened a cookie jar and said basically you can’t put your hand in the jar” (Oncology Nurse Practitioner [2]).

Considering most hospitals and specialist centres are treatment-focused, integrating general practitioners (GPs) was noted as a key opportunity. “Integration with the GP is a really good thing” (Oncology Nurse Practitioner [2]). Reducing corticosteroid usage in chemotherapy regimens was another opportunity. “Some of the opportunities will be around trying to reduce the amount of corticosteroid we use” (Medical Oncologist [5]). Education modules were also noted as an opportunity to enhance oncology HCPs’ knowledge and awareness of sleep. “Something like this would be really helpful… modules on pain… nausea, vomiting… something like this would have a place in there and showcase it for cancer nurses as a priority” (Clinical Nurse Consultant, Cancer Services [1]).

## Discussion

This study explored oncology HCPs’ knowledge, understanding, and clinical management practices of sleep disturbances in individuals with cancer receiving chemotherapy. Four main themes were generated through RTA, including sleep disturbances are underreported and underassessed, poor sleep can be difficult to manage, limited capacity for clinicians to address sleep issues, and opportunities to improve the management of sleep disturbances within cancer care.

The interviewed oncology HCPs perceived sleep disturbances as frequently overlooked by both patients and clinicians and often insufficiently assessed. This represents a significant oversight, as sleep disturbances are associated with greater symptom burden [1] and poorer quality of life [4, 5], which, if left untreated, may worsen and persist into later phases of survivorship [6, 7]. Routine screening and assessment of sleep represent an important opportunity to identify sleep issues in a timely manner and help understand the cause(s) of poor sleep, which can be used to personalise treatments. Clinicians described rarely using sleep PROMs in clinical practice, owing to a lack of resources to treat identified sleep issues, consistent with findings by Jeon et al. [17]. Although barriers at the patient, clinician, and service levels may limit the implementation of PROMs [22], evidence supports PROMs in improving care [23] and represents a key opportunity.

Oncology HCPs described limited control over certain contributors to poor sleep, particularly corticosteroid medications. Where possible, they recommended measures such as morning administration to minimise sleep disruption. Other treatment- and disease-related factors, including fatigue and pain, were also commonly identified as affecting sleep. Fatigue frequently co-occurs with sleep disturbances and can be difficult to distinguish [24], potentially leaving sleep issues untreated, increasing symptom burden and reducing quality of life. When pain impacted sleep, oncology HCPs considered palliative care services effective in their management. Psychological factors, such as anxiety and depressive symptoms, were also recognised but seen as more difficult to address due to limited access to psychological support. Overall, these factors were viewed as challenging to manage, limiting oncology HCPs’ ability to address sleep disturbances effectively.

Verbal advice and pharmaceutical sleep aids were the most common treatments utilised to manage sleep disturbances. Clinicians would discuss sleep hygiene with patients, although recommendations reportedly had varied effectiveness. This aligns with previous work confirming sleep hygiene’s limited effectiveness as a monotherapy [25]. Pharmaceutical sleep aids were commonly prescribed to manage sleep disturbances in this population. Although clinicians were reluctant to rely on benzodiazepines, they were prescribed selectively and on an intermittent basis to mitigate potential side effects and dependency issues. Olanzapine and mirtazapine, which have sleep promoting qualities [26, 27] were viewed as favourable alternatives which may also offer benefits in palliation of other symptoms [28]. Where a symptom (i.e. hot flushes) significantly impacted sleep, clinicians found that treating the symptom was often effective at managing sleep issues. Overall, pharmaceutical sleep aids were perceived to be the most effective treatment in this population.

Several barriers to managing sleep disturbances were reported, most notably time constraints and limited access to supportive care services. Clinicians commented that addressing sleep disturbances takes time, and with competing priorities, sleep was often not prioritised. Likewise, where sleep was impacted by psychological factors, it was perceived to be more difficult to manage due to limited psychological services. Shared care between GPs and oncology HCPs was identified as a key opportunity. Shared care models are associated with greater patient satisfaction, cost savings, and non-inferior outcomes compared to specialist-led care [29]. Interestingly, despite pre-existing clinical practice guidelines specific to sleep disturbances and insomnia in cancer [12, 14, 15], clinicians noted more cancer-specific guidelines would be useful. Improving awareness and uptake of these guidelines amongst oncology HCPs may be a useful target, although this may not be pragmatic considering time constraints and limited access to supportive care services. Furthermore, education modules were also identified as an opportunity to enhance oncology HCPs’ knowledge and understanding of best management practices of sleep disturbances specific to cancer. However, these modules would need to be co-designed with oncology HCPs, along with input from sleep specialists, to ensure the modules reflected best practice and were suitable to the target audience.

Several limitations need to be considered when interpreting our findings. First, participants were recruited locally in Perth, Western Australia. Subsequently, findings may not be reflective of knowledge, understanding, and sleep management practices of oncology HCPs in other states across Australia, nor the world. Second, participants predominantly practiced in a treatment-focused model. Those working in survivorship centres with greater access to supportive services may manage sleep differently. Third, this study was limited to exploring clinical management practices of sleep disturbances during chemotherapy. Although anti-cancer therapies share factors that contribute to poor sleep, each therapy has distinct side effects that may impact sleep differently. Strengths of this study include discussing real-world clinical management practices used by oncology HCPs. Exploring real-world practices, their perceived effectiveness, and barriers to managing sleep disturbances offers insight into opportunities for improvement. Importantly, this study sought perspectives from a medical oncologist and nursing perspective; both HCPs are critical to managing sleep disturbances in this population. Furthermore, RTA is a flexible approach to analysing qualitative data [30]. This approach facilitated the authors to deeply engage with the data and practice reflexivity, whilst acknowledging that their subjectivity influenced the themes generated [30].

## Conclusion

This study offers useful insights into oncology HCPs knowledge, understanding and clinical management practices of sleep disturbances in individuals with cancer receiving chemotherapy. Whilst sleep disturbances were regarded as an important issue, it was perceived to be under-reported and underassessed—in routine care. Knowledge gaps, time constraints and limited access to resources were identified as key barriers in care that limited HCPs ability to effectively manage sleep issues. Opportunities that emerged include enhancing HCPs awareness and knowledge of evidence-based sleep treatments, increased screening and integration of PROMs, greater emphasis on shared care with GPs and improving equitable access to supportive care services. Efforts to implement these opportunities should focus on co-design with relevant stakeholders at all levels (i.e., patients, clinician, service level).

## Supplementary Information

Below is the link to the electronic supplementary material.

ESM1 (DOCX 24.1 KB)

ESM2 (DOCX 16.2 KB)

## Data Availability

The data that supports the findings of this study are available from the corresponding author upon reasonable request.
