# Supplementary material for: Clinical insights into the management of sleep disturbances within cancer care: a qualitative analysis
Source: Support Care Cancer. 2026 Feb 27;34(3):257. doi: 10.1007/s00520-026-10478-4 (PMC12948860; doi:10.1007/s00520-026-10478-4)
Supplement: Supplementary file 2 — (DOCX 16.2 KB) [file 520_2026_10478_MOESM2_ESM.docx]

Oncology Healthcare Professional Interview Guide

Welcome the clinician and explain that the purpose of this semi-structured interview is to gain a better understanding of how sleep disturbances are managed within cancer care.

1. ***Can you please describe your role and responsibilities within cancer care?***
2. Enquire occupation and their role(s) with patients.
3. ***Can you please describe how you identify sleep issues in cancer patients during chemotherapy treatment?***
4. ***Do you have any tools or assessments that you use to assess the severity and impact of sleep disturbances?***
5. ***Currently, what treatments and/or strategies do you use to address chemotherapy related sleep disturbances?***
6. ***Are these effective?***
7. ***What challenges do you encounter in managing sleep disturbances in this population?***
8. ***If able to, how do you overcome these challenges/barries?***
9. ***Removing any challenges and barriers; in an ideal world, how would you best treat sleep disturbances***?
10. ***Do you think there are any opportunities to better improve the management of sleep disturbances in cancer care?***
11. Closing
12. Summarise key points from discussion and provide opportunity for them to share any additional information.
